# 16S rRNA amplicon sequencing of intestinal microbiota in Eurasian Wigeon around Osaka, Japan

**DOI:** 10.1128/mra.00259-24

**Published:** 2024-06-11

**Authors:** Takehiko Kenzaka, Sayaka Nishiguchi, Souko Nishikawa

**Affiliations:** 1 Faculty of Science and Engineering, Setsunan University, Neyagawa, Osaka, Japan; 2 Faculty of Pharmacy, Osaka Ohtani University, Tondabayashi, Osaka, Japan; Loyola University Chicago, Chicago, Illinois, USA

**Keywords:** avian, bird, Eurasian Wigeon, 16S, *Mareca Penelop*

## Abstract

We report 16S rRNA gene amplicon data on the succession of intestinal microbiomes in migratory bird, Eurasian Wigeon (Mareca Penelope) wintering around Osaka, Japan. This study could serve as baseline data for comparison with microbiomes in migratory birds.

## ANNOUNCEMENT

Migratory birds may harbor pathogenic bacteria. There have been cases in Europe where birds have died in large numbers due to fowl cholera bacteria of wild bird origin (1). It is estimated that large amounts of the bacteria travel long distances between Japan and other countries with migratory birds (2), but the actual nature of the microbiome transfer remains unknown.

Eurasian Wigeon (*Mareca Penelope*) breeds in northern Eurasia, including Siberia, and migrates to India, Southeast Asia, East Asia, and North America in winter (3). They inhabit mainly lakes, rivers, and beaches, feeding on plants on the water’s surface as well as on plants on land. The global population of Eurasian wigeons is estimated at 2.8–3.3 million individuals (4).

Recent culture-independent genetic approaches have clarified the phylogenetic diversity and novelty of microbiomes associated with migratory birds (5). Here, we provide baseline data on the succession of the intestinal microbiome of Eurasian Wigeon wintering in the western part of Japan.

A total of 127 fecal samples of *M. penelope* wintering riverside of the Aigawa River (34.841 N, 135.570 E) were collected from the glass in two wintering seasons every month (between December 2017 and April 2018, and between December 2018 and March 2019). The river is one of the major rivers flowing through the northern part of Osaka and the riverside area has been developed for leisurely strolls along the river. Only fresh droppings were collected under conditions where no other birds were present. About 100 mg of fecal samples was subjected to DNA extraction using the QIAamp Fast DNA Stool Mini Kit (Qiagen KK, Tokyo, Japan).

In the first PCR, the V4 region of the prokaryotic 16S rRNA gene was amplified using F515 and R806 primers (6) with the adapter sequences for the second PCR. The PCR products were purified using Agencourt AMPure XP (Beckman Coulter, CA, USA). A second PCR was performed to attach indices and sequencing adapters using Nextera XT Index Kit v2 (Illumina, CA, USA). The indexed amplicons were purified by electrophoresis using E-Gel SizeSelect II Agarose Gels (Thermo Fisher Scientific, MA, USA) and mixed. Paired-end reads (2 × 250 bp) were obtained from a MiSeq instrument (Illumina). Negative control, processed in the same DNA extraction and PCR with DNA-free water, was included. Raw reads were processed in R studio (Version 2023.12.1+402) using DADA2 (v1.30) package (7) following the Pipeline Tutorial 1.16 (https://benjjneb.github.io/dada2/tutorial.html) for quality filtering, denoising, merging, ASV inference, and chimera removal. Amplicon sequence variants (ASVs) were analyzed using the SILVA rRNA database (v138.1) (8). Sample and sequence data summarized for 9 months are presented in Table 1.

**TABLE 1:** Summary of metadata and accession numbers of the 16S rRNA amplicon sequences of fecal samples of Eurasian wigeon, along with ASVs classified against bacterial taxa

Sample name	Collection date	No. of fecal samples	No. of raw read pairs	No. of mapped read pairs	No. of ASVs	No. of unique ASVs	BioSample accessions
1712AI-Hidori-F	December 17	17	786,303	697,680	1,993	850	SAMN40407066
1801AI-Hidori-F	January 18	15	986,744	874,620	2,208	1,058	SAMN40407067
1802AI-Hidori-F	February 18	16	704,666	633,660	1,098	486	SAMN40407068
1803AI-Hidori-F	March 18	16	883,409	789,489	1,952	694	SAMN40407069
1804AI-Hidori-F	April 18	11	1,312,388	1,184,031	2,270	1,094	SAMN40407070
1812AI-Hidori-F	December 18	12	814,319	775,033	1,815	715	SAMN40407071
1901AI-Hidori-F	January 19	9	660,921	637,952	534	89	SAMN40407072
1902AI-Hidori-F	February 19	14	594,334	552,553	2,359	1,042	SAMN40407073
1903AI-Hidori-F	March 19	17	890,540	781,288	1,719	597	SAMN40407074

The amplicon sequencing of 127 samples generated 7,633,624 raw read pairs, of which 6,926,306 quality read pairs were classified into 9,165 ASVs of bacteria, with 39 common ASVs and each uniquely detected ASVs. These ASVs were represented by 28 phyla and 672 genera. Proteobacteria, Bacteroidetes, Firmicutes, and Campilobacterota constituted >90% of the gut bacteria. The data obtained in this study could serve as baseline data for comparison with microbiomes in migratory birds.

## Data Availability

Data are available under BioProject number PRJNA1086667.
